# Consumer Experience of an Australian Multidisciplinary Long COVID Clinic That Incorporates Personalised Exercise Prescription: A Qualitative Analysis

**DOI:** 10.1111/hex.70179

**Published:** 2025-02-28

**Authors:** Tanya Buettikofer, Allison Maher, Veronica Rainbird, Michelle Bennett, Nicole Freene, Imogen Mitchell, Hsin‐Chia Carol Huang, Philip Gaughwin, Mary Johnson, Jenny Paratz, Bernie Bissett

**Affiliations:** ^1^ Canberra Health Services Canberra Australia; ^2^ Faculty of Health, University of Canberra Canberra Australia; ^3^ College of Health and Medicine, Australian National University Canberra Australia; ^4^ The Royal Brisbane and Women's Hospital Brisbane Queensland Australia; ^5^ Griffith University Brisbane Queensland Australia

**Keywords:** consumer experience, exercise, Long COVID, multidisciplinary, rehabilitation

## Abstract

**Background:**

In Australia, Long COVID is prevalent in 5%–10% of COVID‐19 cases. Few multidisciplinary services exist to support recovery from Long COVID.

**Objective:**

To understand the consumer experience and acceptability of a novel Australian Long COVID Recovery Clinic, which incorporates personalised exercise prescription including respiratory and peripheral strengthening and carefully monitored cardiovascular training.

**Design:**

Qualitative study; semi‐structured interviews conducted by a researcher external to the clinic delivery.

**Setting, Participants:**

A convenience sample of participants who have completed the Long COVID Recovery Clinic.

**Main Outcome Measures:**

Major themes were identified by inductive thematic analysis.

**Results:**

Fifteen participants were interviewed. 14/15 (93%) participants described the clinic model as acceptable or highly acceptable. Five core themes were identified, including (1) encouraging staff and light‐filled facilities support recovery; (2) supervised exercise and pacing improve confidence with exercise; (3) peer support and group therapy augments recovery; (4) other services augment Long COVID recovery, and (5) importance of GP involvement in connection with clinic participation. Suggestions for improvement included extending the duration of the clinic programme beyond 2 months, reducing wait times by increasing staffing levels and adjusting the clinic schedule to broaden access options.

**Conclusions:**

The majority of participants found that the Long COVID Recovery Clinic, which incorporates both supervised exercise and pacing, is acceptable and would recommend it to others. From the consumer perspective, the Long COVID Recovery Clinic aids recovery alongside GP management through a combination of peer support and an individually tailored programme.

**Patient or Public Contribution:**

A consumer was a highly valued member of our research team. She has been involved in study design, analysis, and interpretation. She has also been involved in editing the manuscript and provided advice to ensure the language used in the manuscript is sensitive to a consumer audience. As our consumer meets the authorship guidelines, we have included her as an author in this manuscript. We also intend to include our consumers in the dissemination of these results when published (e.g., social media).

**Trial Registration:**

The study was registered with the Australian New Zealand Clinical Trials Registry, ACTRN12622000719730.

## 1 Introduction

Post‐COVID‐19 Condition or Long COVID is defined as symptoms lasting greater than 3 months following an infection with COVID‐19 [1]. In Australia, the prevalence of Long COVID is 5%–10% of COVID‐19 cases [2]. An Australian study investigating consumers who survived an Intensive Care Unit admission for COVID‐19 showed that 39% continued to have symptoms and new‐onset disability at 6 months [3]. Another recent Australian study described the debilitating symptoms and disability which persist in Long COVID [4]. Symptoms most commonly featured dyspnoea (28%) and extreme fatigue (28%) [4], echoing global patterns of this disease burden [5].

Globally, studies have examined the use of pulmonary rehabilitation in supporting recovery from Long COVID [6] and have reported improvements in dyspnoea, physical function and quality of life [7]. However, there are very few publicly funded services in Australia which include a coordinated multidisciplinary team to support recovery from Long COVID.

Current literature about the experience of consumers living with Long COVID largely focuses on their experience in establishing a supportive healthcare team, rather than their lived experience of rehabilitation programmes [8, 9]. Previous studies have also highlighted the psychological consequences of Long COVID such as depression and anxiety [10] and the need for healthcare professionals to validate consumers' symptoms following COVID‐19 infection to optimise care [8, 9]. With these principles in mind, we sought to explore the experiences of consumers engaging with a rehabilitation service specifically optimised to meet the needs of people with Long COVID.

In March 2022, the multidisciplinary Long COVID Recovery Clinic was established at the University of Canberra Hospital. In Australia, this service is provided as part of free public healthcare, and people of all backgrounds are eligible to attend if they meet the clinical criteria. A detailed description of the clinic setting and facilities, inclusion criteria, referral pathways, triage, screening and intervention is available elsewhere [11]. Briefly, the clinic is centred on individualised assessment of needs; personalised prescription of tailored progressive exercise therapy and holistic case management designed to meet consumers' physical, cognitive, psychological and social needs. The health professionals in the multidisciplinary team include a Rehabilitation Medicine Physician, a Post‐COVID Recovery Clinic Coordinator, Physiotherapists, Exercise Physiologists, Occupational Therapists, Allied Health Assistants, a Dietitian and a Social Worker. Consumers attended approximately two sessions per week for up to 12 weeks. The novel aspect of this clinic, compared to others around the world, is an approach to exercise prescription that is more progressive than current guidelines for rehabilitation in Long COVID [12, 13], including strength training of both inspiratory and peripheral muscles. The aim of this study was to understand the consumer experience and describe the acceptability of this novel approach to COVID‐19 recovery in an Australian context.

## 2 Methods

### 2.1 Design

This qualitative study focused on consumer experience using the approach of reflexive thematic analysis [14] to richly understand the lived experiences of clinic participants. Inclusion criteria were adults who attended the multidisciplinary Long COVID Recovery Clinic at the University of Canberra Hospital (aged 18 years or older) who consented to be contacted for the interview. There were no exclusion criteria. We aimed to recruit a heterogeneous, convenience sample of 15 participants from October to December 2022. Consecutive participants who completed the clinic during this time were invited to participate by an investigator (A.M.) during face‐to‐face sessions at the clinic. Interested participants signed a consent form including their preferred method to be contacted for the interview by another researcher who was uninvolved in clinic delivery (T.B.). Interviews continued until no new key themes were identified. The number of participants was guided by the specificity of the study population and the monitoring of the identification of key themes [14]. The Consolidated Criteria for Reporting Qualitative research was used to guide the reporting of the study [15].

### 2.2 Setting

The Long COVID Recovery Clinic is located at the recently built facility, the University of Canberra Hospital, a dedicated, standalone rehabilitation hospital. Access to the clinic is through a general practitioner or health professional referral. Participation in the clinic is free of charge to clients (publicly funded). Clients are screened on referral by a health professional and, if suitable, are added to the clinic waitlist. Clients of the clinic experience coordinated healthcare from a multidisciplinary team including physiotherapists, occupational therapists, exercise physiologists, dietitians and others, with oversight from a rehabilitation physician. Where indicated, specialised screening and exercise testing is provided through a respiratory specialist at Canberra Hospital. The clinical intervention typically includes peripheral muscle strengthening, inspiratory muscle strengthening, whole‐body cardiovascular exercise, cognitive rehabilitation, dietary advice, health coaching and education.

### 2.3 Theoretical Perspective

We approached data collection and analysis from a social constructionist perspective, based on the theory that knowledge is developed in a social context rather than individual psychologies [16]. Experiences and meanings are produced through human interaction and culture and influenced by language [17]. This perspective is relevant to this study as the model of the Long COVID Recovery Clinic involves group exercise and education where participants interact with one another and staff, influencing their experience. As the study examined the consumer experience, a relativist ontology was assumed. In addition, the authors recognise that this analysis represents one of multiple possible interpretations of the data and is necessarily informed by the perspectives and subjectivity of the researchers [14] who have lived experience working in health care and clinical research.

### 2.4 Semi‐Structured Interviews and Demographic Data

The interview format was offered via either online video conference or face‐to‐face lasting 30 to 60 min. Semi‐structured interviews were conducted by the lead investigator (T.B., physiotherapist and PhD candidate). Participants were informed that the interviewer was a health professional independent of the delivery of the COVID clinic. Interviews followed a topic guide (Appendix) of open‐ended questions developed by two investigators (T.B. and B.B.) to explore participants' experience and acceptability of the clinic. This topic guide was informed by the existing literature [18, 19], with an awareness of the importance of peer support and validation of experiences [8, 9]. Each interview was audio recorded, professionally transcribed verbatim and de‐identified for confidentiality and anonymity. Transcripts were sent to participants, and 14 confirmed the transcripts as an accurate representation of the interview and meaning. Two participants provided eighteen points of clarification.

Participants' demographic data were extracted from the medical record.

### 2.5 Data Analysis

Analysis followed the six phases of thematic analysis [20] within a social constructionist paradigm. A latent approach was used, which considers assumptions and ideas underlying the data [20]. Investigators interpreted the data with respect to a broader research question of examining the clinical benefit of a follow‐up service for those with Long COVID.

All researchers involved in the thematic analysis were not involved in clinic delivery. Two investigators (T.B., B.B.) independently analysed transcripts 1–5 using an inductive approach and manually generated initial themes through open coding followed by focused coding. Sub‐themes and key themes were generated and discussed until a consensus was reached. The lead investigator (T.B.) analysed the remaining transcripts. Sub‐themes and key themes from the entire data set were then discussed with experienced researcher B.B. and a third investigator (N.F., experienced qualitative researcher, physiotherapist, PhD) until a consensus was reached.

### 2.6 Ethics Approval

Ethics approval was obtained from the Human Research Ethics Committees of both the University of Canberra (#11722) and ACT Health (ACT Reference 2022.LRE.00038, REGIS Reference 2022/ETH00369).

## 3 Results

We identified five core themes in which consumers describe the Post COVID Recovery Clinic, including the importance of validation of their condition by staff and peers; the impactful nature of the facilities in which the care was provided; the enhancement in exercise confidence resulting from supervised exercise; the value of peer support during recovery; the need for additional services to augment the clinic model and the crucial role of the General Practitioners in coordinating their care before, during and after their clinic experience.

### 3.1 Characteristics of Participants

Fifteen participants were interviewed (14 videos, 1 face‐to‐face), and three participants were invited but did not respond. Table 1 outlines the participant characteristics. Characteristics of non‐responders did not differ from responders in terms of the criteria listed below. All participants had completed the main clinic programme within the previous month and were aged between 23 and 63 years. The majority were female. There was a difference in the number of pre‐existing conditions, work status and wait times from referral to initial assessment between the participants (Table 1). No participants identified as being of Aboriginal or Torres Strait Islander background, and all spoke English.

**Table 1:** Participant characteristics.

Participant	Age (years)	Sex	Country of birth	Pre‐existing conditions	Work status before COVID‐19 infection	Wait time from referral until initial assessment (days)
P1	60	F	Australia	> 1 LTC	FT	36
P2	56	F	Australia	> 1 LTC	Employed (FT/PT unspecified)	8
P3	51	F	Australia	None	FT	82
P4	34	M	Australia	None	PT	111
P5	47	M	Australia	> 1 LTC	Employed (FT/PT unspecified)	71
P6	56	F	England	> 1 LTC	FT	33
P7	57	F	Australia	None	Employed (FT/PT unspecified)	98
P8	35	F	Australia	> 1 LTC	FT	70
P9	63	M	Australia	None	Retired	36
P10	60	F	New Zealand	> 1 LTC	Employed (FT/PT unspecified)	125
P11	58	F	Australia	> 1 LTC	Employed (FT/PT unspecified)	99
P12	43	F	Ireland	1 LTC	PT	68
P13	34	F	Australia	1 LTC	FT	111
P14	23	M	Australia	> 1 LTC	FT	113
P15	26	F	Australia	> 1 LTC	FT	141

Abbreviations: F, female; FT, full‐time; LTC, long‐term condition; M, male; P, participant; PT, part‐time.

### 3.2 Acceptability and Overall Experience

There was broad acceptability of the clinic with 14 participants reporting the clinic to be acceptable and stating that they would recommend it to others. Overall participants described positive experiences in the clinic and some reflections were specific about physical aspects of their recovery.

‘I'm so much better having gone to the COVID clinic. It's not just learning to manage my exercise. I think my metabolism and my muscles and my heart/breathing coordination has improved massively’.

(Participant 7, 57 F)

The one participant who did not find the clinic acceptable reported frustration with the lack of access to experimental drugs and therapies.

### 3.3 Core Themes

Five core themes were identified as outlined below.

#### 3.3.1 Theme 1. Encouraging Staff and Light‐Filled Facilities Support Recovery

Most participants commented on the encouraging staff being invaluable to their experience at the clinic and supportive of their recovery. Participants mentioned the staff valued their questions and provided helpful and thoughtful responses.

‘The enthusiasm and the knowledge of the instructors [staff], … they were supportive and encouraging and enthusiastic … If you ever asked a question of them, it was considered and answered. You weren't left hanging’.

(Participant 9, 63 M)

It was highlighted by many participants that the clinic staff frequently validated their symptoms. This was not always an expected outcome of attending the clinic but was appreciated. Validation of their symptoms was perceived as comforting. Once they felt validated, participants were able to shift their focus to understanding how to manage their symptoms and incorporate daily tasks and exercise around their symptoms.

‘Yes, I think it was really helpful. It made me feel like I wasn't going crazy, it wasn't just something that was in my head that I was being lazy. I didn't try hard enough. It validated the fact that I was still recovering from the virus, and I wasn't imagining it, which is what I needed at the time’.

(Participant 12, 43 F)

A consistent reflection was that the physical facilities also aided recovery. The new purpose‐built facilities, incorporating natural light, provided a calm environment for participants as a foundation for their recovery.

‘I just like the place. There is a toilet around every corner … the main centre hub there with the coffee shop and with the large ceiling and the sun coming through, the natural light. … if you were trying to do this in the old 1970s buildings, it would just feel like you're in a detention centre. Like this place allows you to relax a little’.

(Participant 5, 47 M)

#### 3.3.2 Theme 2. Supervised Exercise and Pacing Improve Confidence With Exercise

Most participants reported that a combination of exercise and pacing was crucial to improve confidence in their daily activities. Some participants mentioned that supervised and individually progressed exercise was crucial to their rehabilitation progress. Others found that they could incorporate pacing strategies into their daily activities outside of the clinic.

‘Exercising there was easy, pacing yourself there was easy, at home it was much more difficult to get the pacing right’.

(Participant 9, 63 M)

‘Sort of gave you confidence that actually you were OK, you didn't have to worry too much that you weren't putting yourself at risk by doing vigorous exercise, etcetera’.

(Participant 10, 60 F)

Some participants reflected that they did not have an accurate understanding of their exercise capability before attending the clinic. Participants linked this to the post‐exertional malaise felt after either activities of daily living or when attempting exercise.

‘… I think they really helped with learning about that post‐exertional malaise and trying to learn to pace myself and not do too much all the time and accept the fact that this is what I was like for now. And I think that's what I needed at the time was someone to tell me that it's OK to feel tired, or it's OK to have a nap … as opposed to try and push myself all the time’.

(Participant 12, 43 F)

‘I guess I just didn't notice how intolerant of exercise I had become, because I didn't really try to do any sort of physical activity, other than just your day‐to‐day stuff’.

(Participant 12, 43 F)

‘I hadn't worked out how to exercise. I just kept trying to exercise and falling in a heap the next day’.

(Participant 7, 57 F)

#### 3.3.3 Theme 3. Peer Support and Group Therapy Augments Recovery

Peer support was a key theme identified by most participants. This support was not always a predicted outcome of attendance at the clinic.

‘Meeting other people that are going through the same thing … I was surprised how much I got out of that. It wouldn't have been a reason to join. I wasn't expecting that that would be really useful. But I think that was really useful’.

(Participant 7, 57 F)

Participants felt validation from each other. This assisted with their emotional recovery and building confidence. Several participants highlighted the importance of receiving validation from someone who could sympathise with their condition. Others recognised the importance of group discussion in providing emotional support.

‘Interaction with other COVID patients …those who are going through the same things that I was going [through] helped me mentally and emotionally not just empathise, but also sympathise with what I was going through and what they were going through’.

(Participant 6, 56 F)

‘You come in here and you see other people with the same symptoms, you know you're like well, thank God, I'm not the only Looney Tune on the planet’.

(Participant 5, 47 M)

Participants highlighted peer support in the form of constructive peer pressure. A driver to attend the exercise classes was not letting others down by not being there. Within the class, peers would motivate each other to continue their exercise programme or complete more exercises. They also highlighted the positive effect this experience had on their mood.

‘You'll let people down if you don't go. … Doing it in an environment with other people that were laughing, because they said oh God, I can't even do ten today. Come on you lazy chap, you'd egg each other on. And we'd walk out and every single time we'd all be smiling’.

(Participant 2, 56 F)

Several participants met independently of the programme after the exercise classes, for example, Facebook groups and coffee catch‐ups. They also expressed that this could be useful for future participants. In contrast, some people perceived that pre‐existing Facebook groups outside of the Long COVID Recovery Clinic seemed to ‘wallow’ in their condition (Participant 1, 60 F) and were therefore unhelpful for recovery.

#### 3.3.4 Theme 4. Other Services Augment Long Covid Recovery

Some participants spoke about ongoing symptoms and felt that they were not completely ‘fixed’ after being discharged from the Long COVID Recovery Clinic. Participants recognised that improvements had been made during their clinic experience whilst still perceiving the need for further recovery

‘… the brain fog diminished, the medication for depression has probably had a big part to play with the depression. I think my biggest problem remains that … I can walk, I can cycle, I can garden, I can do most things, there just seems to be some problem still with jogging or running’.

(Participant 9, 63 M)

Other services were used to augment the clinic. These services included private occupational therapy, and one participant specifically mentioned the prescription of off‐label medications from specialists who were willing to prescribe these. Other services, which were accessed outside of the usual clinic structure, included dietitians and psychologists. Also, hydrotherapy was a service that participants wished to access to augment their recovery. Some participants acknowledged that the cost was prohibitive for what they would like to do.

‘I'm a single income paying my mortgage, you know, and I can't afford to pay $20 a session for a half an hour pool session’.

(Participant 6, 56 F)

Several participants mentioned using their own resources whilst waiting for the Long COVID Recovery Clinic to open or whilst attending the clinic. These included pain specialists, private swimming lessons and gym memberships. Participants highlighted that knowing which services to access was challenging, but they felt the need to take action to support their rehabilitation while waiting for the clinic.

‘I ended up going to see a pain specialist and she's been fantastic and actually has started to make some difference. But that was never mentioned to go to see a pain specialist. It was actually my doctor who suggested going to see a pain specialist’.

(Participant 8, 35 F)

#### 3.3.5 Theme 5. Importance of GP Involvement in Connection With Clinic Participation

The general practitioner (GP) was seen as fundamental to accessing the clinic. Participants highlighted that the clinic operated alongside the GP, therefore good communication between these services is essential to optimise client improvement.

‘…you need a GP to give you the springboard in [to the programme]’.

(Participant 1, 60 F)

‘… go through their GP and make sure their GP keeps in touch with the clinic and also to make sure they have the chance to voice how they feel right from the word go’.

(Participant 6, 56 F)

In addition, participants recognised that two‐way learning occurs between the GP and participants, particularly with a new condition with new referral pathways.

‘I think for my GP, I think it was really helpful for her to be able to say here's something I can help you with, refer you on. And I actually went back to her, and she said I've taught her so much stuff about Long COVID and the process’.

(Participant 12, 43 F)

Another recurring issue was the length of the waitlist with some participants needing to wait over 6 months for an initial appointment. Beyond these themes, there are numerous suggestions to improve the Long COVID Recovery Clinic (Table 2). Although beyond the original aims of this study, we include these spontaneous suggestions to maximise the value of consumer voice.

**Table 2:** Suggestions for improvements to the Long COVID Recovery Clinic.

Suggestion	Quote
Consider changing the name from COVID ‘*Recovery*’ Clinic	‘When you hear the name Long COVID Recovery Clinic you instantly think they may not cure me, but they're going to be definitely do something … you'd expect some sort of form of recovery given the name of the service’. (Participant 5) ‘I'm graduated but like I'm also not recovered by any means’ (Participant 4) ‘I think the only thing is my disappointment in not doing that full programme of activities to be able to understand, you know well it says eight sessions, I've done five, I'm owed three, maybe that last three could have pushed me further to understand more about the lung issues that I'm having’. (Participant 6)
Clinic follow‐up should be longer due to persistence of some symptoms	‘So whether it's having a check in, in a months' time and then 3 months. Or I don't know if the programme would benefit from being any longer than that 8‐week period, but I think maybe some of that support when you're at home doing the exercises, whether that can be added on there. I think that would be really good. I know I've spoken to a few who were in my initial class, that felt a bit, what's the word? A bit, not dumped, but I can't think of the word, a bit left on the side, forgotten about. And felt that they hadn't really achieved what they wanted to, I suppose, and didn't really have any warning that it was ending.’ (Participant 8) ‘… when I finish the 8‐week exercise program, they'll give you exercises to take home, and then they're like OK, we're done. I feel there was no explanation before that… I think they need to come up with something so it's not just cut and dry. Whether that's check in with people a month later and seeing if they need to adjust their working from home exercises or I think there needs to be something like that. they've acknowledged that it's a long road, and eight weeks isn't really a heap. And I know it's the plan to be able to take this to home, but I think there needs to be a bit more follow‐on’. (Participant 8)
Schedule of clinic classes requires diversity of options	‘I had problems getting there at 3 o'clock’. (Participant 6) ‘[would be good to] have the option of different [class] times. Because I think a lot of the group classes were like three in the afternoon’. (Participant 12) ‘I did see the OT quite a few times and she had, I think they had run maybe like a memory or something like that group, that I also, she said that would be really good for you to go to, but again it was at a time where I couldn't attend. So I guess it was just those sorts of things would have been good to go to’. (Participant 12) ‘If it was later in the afternoon 3:30, probably closer to four, especially it its only going to be 45 min … even if they did a split session where you had those who could come in the mornings, and then you had a second shift that came in from 10 til six’. (Participant 6) ‘because of the kids, they had group exercise classes, but I couldn't attend those, but they were very flexible with helping me, they had an allied health professional that would meet with me at a different time, because the group classes were at 3 o'clock, which is the time I pick my kids up from school. So they were really accommodating in trying to help me fit in around them’. (Participant 12) ‘one thing that I felt would have helped is if they had more group classes. I know they did have some, but unfortunately I couldn't attend them at those particular times. But I think because it just feels like such an isolating thing. People just don't get it, lots of people have had Covid, and they're fine, but it's really hard when people think, what's wrong with you, why do you still feel sick? I think it's one of those really isolating things. I guess it's the same with any chronic illness, where it's just nice to meet someone that you know you're not going crazy, these things actually have affected your body, and you're just not the same person that you were. I probably think that. I think that would have been good, if I could have connected with other people in the same situation’. (Participant 12)
Staff could provide more clarity around approach used i.e. not experimental drugs	‘Some Long Covid clinics you hear about they're testing drugs and they're doing that, and I guess that would be another reason for the University of Canberra Long Covid clinic to clearly state at each session what they're on about. Because they're not on about testing drugs or that sort of stuff’. (Participant 9) ‘I think they need to provide a bit more of a description of what the program is going to entail. I feel that's lacking a little bit’. (Participant 8)
Potential benefit of expanding programmes to include hydrotherapy	‘My GP said, “you need to do some aqua therapy,” and there was no … it was very much like push, it was like no, no we can't provide that’. (Participant 6)
Clinic to include other conditions with persistent symptoms, not just COVID‐19	‘I just hope it keeps happening for people. Or I hope it expands or something, because it really did make such a huge… I don't really know anyone who cropped (sic) it as hard as I did, but even just small things, especially initially in the first few months after having Covid the first time, a lot of people did really cop it, just fatigue. Because it was so common, we're so exposed to things like influenza and stuff like that, we don't really get these long, long‐run viruses anymore. A new virus type, it can take a long time to come back and that's kind of normal. But we're just not used to that, we're not equipped with how to deal with that. So I hope it keeps running, and the accessibility and availability increases and continues’. (Participant 14)
Potential value of involving an immunologist in the clinic/blood tests	‘I'm getting a lot out of [the clinic] so I wasn't particularly… but even from a research point of view, there's probably something, like a blood draw or something. That's probably my only real gap, is that one, in my first day experience. I feel like this should be of interest to you, but OK’. (Participant 14)
Need to improve the quality of the education sessions	‘I just don't think the education sessions are working … if the speaker is reading from the notes, that encourages the group to read from the notes, but that might actually make their attention between the notes and speech quite tricky’. (Participant 7) ‘Would be good if each time there's a speaker in the education sessions, they could introduce themselves … where you work, maybe even what's their experience with COVID, why they're working in the Long COVID clinic, just so that we've got some sort of rapport with them’. (Participant 7)
Need to improve the quality of handouts and consider online options	‘… there's a lot of mucking around with printing it each day and passing it around which just seems a little bit inefficient. But I also think an online version at home, so people can look it up at home as well. … If you just had a booklet with all the handouts in it… the other beauty of if it was online at home, then the references, because most of the references are links the you could just click straight into the link’. (Participant 7) ‘Sometimes they'd say things like don't do question three, it doesn't make sense. OK, well why are you giving it to us? Didn't you proof read it?’ (Participant 9)
Delays in accessing therapy reflect need to increase staffing	‘Staffing issues … I would have had an OT 3 months ago’ (Participant 5) ‘They've got limited staff … but I would be happy to put my hand up and be a helper’ (Participant 6) ‘There's a doctor, an EP and a physio and they're clearly overwhelmed. And they're doing the best they can for the lives of the people who were there, but they clearly don't have the resources that they need. I was on a waitlist for an OT as well, and it just never happened because they told me the waitlist was too long’. (Participant 13) ‘It took me 6 months to get into the clinic and then I saw them, my first appointment was in September so that's when they put me on the waitlist for the OT. I hadn't heard from them about the OT and when I went back in, they just said, it's just overwhelmed’. (Participant 13)
Consider incorporating advice around chronic pain	‘I feel that's something that they need to explore and address with people who are coming through with chronic pain relating to Covid, that there needs to be another element there that needs to be added on’. (Participant 8)
Preserve the free access to the clinic to maximise accessibility	‘I mean, accessibility obviously, it was a service that didn't have a cost so it was something I didn't have to worry too much about. That took the pressure off. I was more willing to go and do something about it as well’. (Participant 14)

## 4 Discussion

This study explored the consumer experience and acceptability of a novel approach to COVID‐19 recovery that includes progressive exercise as a core feature.

Participants found the clinic positive insofar as the staff validated their symptoms and conditions; they also enjoyed the new physical facilities with natural light and tall ceilings (theme 1). The importance of validation by healthcare professionals has been described in consumers living with depression [10, 21]. Validation of symptoms by health professionals is important given the stigma associated with Long COVID [22, 23, 24], which would likely affect consumers' motivation to access healthcare and to receive treatment for their symptoms. The positive effect of validation found in our study is a valuable insight, as there is a lack of studies investigating Long COVID clinics in Australia that describe the consumer perspective, and cultural norms and attitudes about validation from health professionals may vary around the world. The importance of validation is also relevant to future service design and health professional education in managing Long COVID. Another insight relevant to future service design is the recurrent theme that consumers perceived that natural light assisted with recovery. This is consistent with other studies that showed that natural light enhances attention [25] and is key in the healing process of consumers in healthcare facilities [26].

Participants had a positive perception of exercise and highlighted pacing as a key factor (theme 2). This finding is consistent with the literature on tailored exercise training in consumers with Long COVID [27] and pulmonary rehabilitation programmes [28, 29].

Peer support and group therapy were fundamental for recovery (theme 3). Participants found that validation from others was supportive of emotional recovery. This is well documented in pulmonary rehabilitation literature where peer support is identified by both participants and healthcare workers as an enabler for recovery [30, 31].

The complexity of COVID symptoms meant other services were needed to enhance recovery (theme 4). Whilst this may be perceived as a shortfall of the COVID recovery service, this is consistent with other established services, for example, cardiac rehabilitation, which is considered complementary to the role of the GP and Cardiologist [32].

Participants identified strategies for improvement to the clinic, as outlined in Table 2. Whilst some of these changes can be actioned easily, others are beyond the scope of the clinic (e.g., experimental drug trials). Participants highlighted low staffing levels and excess waiting time as a major concern. The issue of wait times is consistent with other literature showing that prolonged wait times for cardiac rehabilitation reduce uptake [33]. Conversely, participants acknowledged that the Long COVID recovery clinic is accessible due to it being a free service and feel that it should continue as such. It was also identified that improvements to the class schedule could help those working full‐time or those with school‐aged children to attend, which would enhance equity.

Due to the incomplete nature of recovery from Long COVID (theme 4), the General Practitioner (GP) remains the cornerstone of the healthcare team. Participants highlighted that the GP enabled them to attend the COVID Recovery Clinic and supported recovery beyond the clinic (theme 5). This is consistent with other literature, which identifies the importance of GP support to augment the recovery of those with Long COVID [8].

The clinical implications of the research findings are summarised in Box 1. While the specific results of this study may be unique to this single centre, it is likely that the insights gleaned could be used in the development of other Long COVID recovery programmes around the world. Another consideration is that the prevalence of Long COVID may change in coming years. Therefore it is possible that current services focusing on Long COVID may be absorbed into other multidisciplinary rehabilitation services, for example, intensive care unit follow‐up clinics.

Box 1. **Clinical implications of the research findings**

**Clinical implications**

- The COVID Recovery Clinic was acceptable and should continue.
- Peer support group classes could further facilitate recovery.
- Due to the incompleteness of Long COVID recovery, other services are required as an adjunct to the clinic.
- Key improvements required: extend clinic follow‐up timeframes, schedule classes to facilitate attendance of participants and increase staffing to reduce wait times.

### 4.1 Strengths and Limitations

The social constructionist theoretical approach was a strength of the study, as it allows for the possibility that individuals construct their own truth, which is not an objective observation [34]. Rather, their perception of truth is the result of social processes and their interactions. Our in‐depth interviews created space for participants to share their reflections on their experiences of the clinic and thus construct meaning. Whilst attending the Long COVID Recovery Clinic, consumers interacted with each other and health professionals and met outside of the clinic. These interactions also provided a space to reflect on their experience, develop their perception of the clinic and influence their response to questions. We also acknowledge the reflexivity of the research team and perceive that the clinical experience of the researchers was a strength of our analytical approach, as the research team was able to accurately interpret the language and context described by the participants. Furthermore, having interviews conducted by a researcher not involved in clinic delivery was likely to have elucidated authentic answers from the participants, regardless of any perceived fondness or loyalty to the staff delivering the intervention in the clinic.

While our relativist approach allowed individuals to construct their own meaning and describe their unique experience of the clinic, the reflexive thematic analysis revealed several consistent themes across the participants. Contextualisation of these themes could inform the future operation of this clinic and may be useful to other clinics around the world.

There were several limitations in the study. The analysis included assessing the data across participants; however, a second assessment was not completed across each question due to schedule limitations. This may affect the richness of the data collection [14]. While acceptability was captured, this analysis does not provide any information regarding the sustainability or feasibility of the clinic model. Further limitations of the study are that the findings are not representative of the experience of Long COVID in those with an Aboriginal and/or Torres Strait Islander background of a metropolitan population. This is likely a result of the consecutive sampling methodology and the relatively small sample used in a region where only 2% of people identify as Aboriginal and/or Torres Strait Islander [35]. This lack of representation is relevant in Australia due to the ongoing health disparity between Aboriginal and/or Torres Strait Islander people compared with nonindigenous people [36, 37, 38, 39, 40] and is an important area for further research as their experience of healthcare is likely to be different [36]. A final limitation is that memory issues were identified during some interviews, which may affect the reliability of responses to questions. This remains a challenge of qualitative research in participants with Long COVID.

## 5 Conclusion

Most participants found the clinic to be acceptable and would recommend it to others. The Long COVID Recovery Clinic aids recovery alongside GP management through support from peers and staff at the clinic, through an individually tailored approach that included personalised exercise prescription and validation of the consumers' experiences. Key considerations for services for those recovering from Long COVID include to continuing to support it in a public setting, incorporating peer support group classes, scheduling classes to facilitate the attendance of participants and maintaining strong links with adjunct services to enhance recovery. Future research could explore the consumer experience of Long COVID recovery in those with an Aboriginal and/or Torres Strait Islander background, as their experiences may be different from those we have described. In addition, the voice of the consumers from this study should inform future service delivery and may be relevant beyond the Australian context.

## Author Contributions

**Tanya Buettikofer:** conceptualisation, formal analysis, investigation, methodology, project administration, visualisation, writing – original draft preparation, writing – review and editing. **Allison Maher:** conceptualisation, project administration, resources, writing – review and editing. **Veronica Rainbird:** resources, writing – review and editing. **Michelle Bennett:** conceptualisation, writing – review and editing. **Assoc Prof Nicole Freene:** methodology, resources, formal analysis, supervision, validation, writing–review and editing. **Prof Imogen Mitchell:** writing – review and editing. **Dr Hsin‐Chia Carol Huang:** funding acquisition, project administration, writing – review and editing. **Assoc Prof Philip Gaughwin:** project administration, writing – review and editing. **Mary Johnson:** funding acquisition, project administration, writing – review and editing. **Prof Jenny Paratz:** supervision, writing – review and editing. **Prof Bernie Bissett:** conceptualization, formal analysis, funding acquisition, investigation, methodology, project administration, resources, supervision, visualization, writing – original draft preparation, writing – review and editing.

## Conflicts of Interest

The authors declare no conflicts of interest.

## Data Availability

The data are not publicly available due to privacy and ethical restrictions. Deidentified data supporting this study's findings are available from the corresponding author on reasonable request.

## 1

**Topic guide**

**Interview Topic Guide: Acceptability of the Covid‐19 Recovery Rehabilitation Service:**

*We are about to begin the interview for the research project **Patient Acceptability of the COVID‐19 Recovery Rehabilitation Service**. Before we begin, please note that the interview is being recorded for transcription purposes. You may pause the recording at any time or withdraw consent to be recorded at any time. You may also ask for the recording to be deleted at any time*.

*Your participation in the study is completely voluntary and you may withdraw at any time*.

Interview Questions:

1. Can you remember what your expectations of the COVID‐19 Recovery Rehabilitation Service were? And do you think they were met?
2. What was/is the main problem that the clinic helped you address?
  a. (e.g. prompts physical exhaustion, breathlessness, depression, etc)
3. Did you feel that now, the main problem has improved?
  a. If yes, what made the biggest difference?
  b. If no, any ideas what the barriers have been?
  c. Prompt: or did the problems change over time?
4. What were the most useful aspects of the COVID‐19 Recovery Rehabilitation Service for you?
5. Where are the gaps in the COVID‐19 Recovery Rehabilitation service from your perspective?
6. What changes would you recommend to help improve the COVID‐19 Recovery Rehabilitation Service for future patients?
7. Overall, was the service acceptable to you?
  a. Would you recommend it to other people with Long COVID‐19?
    i. If not why not?
8. Do you have any issues or concerns that have not been addressed by the service?
  a. If so, do you have a plan how to manage these issues from here?
9. Is there anything else that you would like to comment on about your experience with the COVID‐19 Recovery Rehabilitation Service?
